# Potential contributors to low dose methotrexate toxicity in a patient with rheumatoid arthritis and pernicious anemia: case report

**DOI:** 10.1186/s41927-020-00175-y

**Published:** 2021-02-12

**Authors:** Miguel A. Jara-Palacios, William Chun, Nomi L. Traub

**Affiliations:** Department of Internal Medicine, WellStar Atlanta Medical Center, Atlanta, GA USA

**Keywords:** Low dose methotrexate toxicity, Vitamin B12 deficiency, Rheumatoid arthritis, Sulfasalazine

## Abstract

**Background:**

Low dose methotrexate toxicity rarely occurs, but may present with severe complications, such as pancytopenia, hepatotoxicity, mucositis, and pneumonitis. Known risk factors for methotrexate toxicity include dosing errors, metabolic syndrome, hypoalbuminemia, renal dysfunction, lack of folate supplementation, and the concomitant use of drugs that interfere with methotrexate metabolism. Vitamin B12 deficiency leads to megaloblastic anemia and may cause pancytopenia, but its role in methotrexate toxicity has not been described.

**Case presentation:**

We present a case of a patient with rheumatoid arthritis who was admitted with febrile neutropenia, pancytopenia, and severe mucositis, likely secondary to low dose methotrexate toxicity. She had multiple factors that potentially contributed to the development of toxicity, including concurrent sulfasalazine use for rheumatoid arthritis. An evaluation of the patient’s macrocytic anemia revealed pernicious anemia. The patient’s illness resolved with cessation of methotrexate and sulfasalazine, leucovorin treatment and vitamin B12 repletion.

**Conclusions:**

This case illustrates the multiple factors that may potentially contribute to low dose methotrexate toxicity and highlights the importance of testing for vitamin B12 deficiency in rheumatoid arthritis patients with macrocytic anemia. Addressing all the modifiable factors that potentially contribute to low dose methotrexate toxicity may improve outcomes.

## Background

Methotrexate (MTX), a folate antagonist, is one of the disease-modifying antirheumatic drugs (DMARD) commonly used in low doses (ranging from 10 to 25 mg weekly) to treat rheumatoid arthritis (RA) and other immune-mediated chronic inflammatory diseases. MTX is generally well tolerated, cost-effective, and highly efficacious at inducing and maintaining remission [1]. However, use-limiting toxicity may occur, more commonly with high doses than with chronic low doses [2, 3]. MTX is typically administered in conjunction with folic acid to decrease the risk of toxicity which can manifest as hematologic, hepatic, pulmonary, infectious, and mucocutaneous adverse effects [4, 5]. Until recently, the rate of adverse effects with low dose MTX had not been precisely determined due to limited data mainly derived from observational studies, case series and case-reports [6, 7]. A recent randomized, double-blind, placebo-controlled trial of low dose MTX in patients without known systemic rheumatic diseases showed that low dose MTX was associated with small to moderate risks of adverse events over a median follow-up of 23 months. The hazard ratio for all adverse events in patients on low dose MTX compared to placebo was 1.17 (95% CI 1.10–1.25) [8].

The antineoplastic effect of high dose MTX results from its interference with folate metabolism. Cancer cells require large amounts of reduced folate to maintain purine and pyrimidine synthesis. Methotrexate competitively inhibits dihydrofolate reductase (DHFR), thereby depleting reduced folates. This leads to a cessation of DNA synthesis and cell cycle arrest in the S phase with eventual cell apoptosis. Very high levels of MTX are necessary to achieve this competitive inhibition of DHFR. The exact mechanism of action of low dose MTX remains incompletely elucidated. Interference with folate-dependent purine and pyrimidine synthesis seems unlikely as the major mechanism of action since administration of folic acid does not result in the loss of clinical effects. Mechanisms proposed to explain the effects of MTX in RA include antagonism of folate-dependent processes, stimulation of adenosine signaling, inhibition of methyl-donor production, generation of reactive oxygen species, downregulation of adhesion-molecule expression, modification of cytokine profiles, and downregulation of eicosanoids and matrix metalloproteinases [9].

We report a case of a patient presenting with pancytopenia and severe mucositis as signs of low dose MTX toxicity. She had multiple possible contributing factors for developing toxicity. We present it to raise awareness of the rare, but potentially severe toxicity from low dose MTX and to outline the risk factors for severe toxicity. This case highlights the complexity of teasing out the true cause of low dose MTX toxicity in complicated patients and addresses the potential contribution of vitamin B12 deficiency to its presentation.

## Case presentation

A 66-year-old African American female presented complaining of five days of difficulty swallowing, swelling of the back of her tongue, painful bloody oral white plaques, and cough productive of blood-streaked sputum. Associated symptoms included chills and the onset of right hand swelling and pain of the right knee.

Her past medical history included rheumatoid arthritis, obesity, type 2 diabetes mellitus, crystal-proven gout, vitamin D deficiency, stage III chronic kidney disease, macrocytic anemia, hypertension, and two episodes of tonsillitis treated with antibiotics in the last two years. She had been diagnosed with RA (ANA+, RF+, CCP-) two years earlier and treated with renally dosed MTX (10 mg) weekly and daily folic acid supplementation with a 1 mg dose. Sulfasalazine 500 mg every 12 h had been added four months prior to presentation to improve disease control. The patient reported taking her last dose of MTX two weeks prior to presentation—she had been advised by a pharmacist to hold MTX when she began a course of amoxicillin clavulanate for a toenail infection. Other medications included metformin, glipizide, hydrochlorothiazide, carvedilol, amlodipine, clonidine, hydralazine, allopurinol, and vitamin D. She denied smoking, alcohol use, or use of recreational drugs.

On physical exam she had a temperature of 38 °C (100.4 °F), a heart rate of 124 beats per minute, and relative hypotension with a blood pressure of 101/77 mmHg. Physical exam revealed dry mucous membranes, severe mucositis, oral candidiasis, active synovitis of the right wrist, and right knee tenderness. She was awake, fully oriented, and had no neurological symptoms.

Laboratory work-up showed pancytopenia, macrocytosis, acute kidney injury, a high serum folate level of 20 ng/ml and a vitamin B12 level of 127 pg/mL (Table 1). Right knee arthrocentesis revealed urate crystals. On admission, medication toxicity was suspected, and methotrexate, allopurinol, sulfasalazine, and metformin were held, while folic acid and vitamin D were continued. She was treated with fluids and broad-spectrum antibiotics for tonsillitis due to febrile neutropenia. Prednisone treatment for a gout flare was initiated. During this hospitalization she was additionally diagnosed with pernicious anemia (positive intrinsic factor antibody and negative parietal cell antibody) and received vitamin B12 injections (1000 μg daily for a week) for repletion.

**Table 1:** Baseline and trend of the patient’s laboratory work-up

	Baseline	Day 1	Day 4	Day 7	Day 13	2 months follow-up	8 months follow-up	Reference range and units
WBC	5140	1450	1920	2260	13,990	10,320	7650	3500–10,500 (cells/ul)
Absolute neutrophils	2990	420	840	990	6440	7970	5550	1700–7000 cells/ul
RBC count	2760	2700	2910	3090	2810	2980	3720	3900–5030 cells/ul
Hemoglobin	9.7	9.4	9.9	10.2	8.9	9.7	11.3	12–15.5 g/dl
MCV	105	104	104	102	110	109	105	82–98 fl
Platelets	161	82	49	311	688	150	156	150–450 units/ul
Creatinine	1.17	1.65		1.06	1.01	1.09	1.58	0.5–0.9 (mg/dl)
BUN	21	65		21	22	16	33	8–23 (mg/dl)
GFR	56	37		63	67	61	39	ml/min/1.73 m2
Reticulocyte count		0.36		2.41	4.44			0.6–1.8%
Serum folate		20						5–24 (ng/ml)
Vitamin B12		127						180–914 (pg/ml)
Vitamin D,25-OH		7						30–100 (ng/ml)
LDH		426						140–271 IU/l

Methylentetrahydrofolate reductase genetic test: Negative for the variants C677T and A1298C

Throughout the patient’s hospital course, no fevers recurred. Blood and synovial fluid cultures were sterile. No focus of infection was identified other than oral candidiasis, which was treated with fluconazole for 7 days. Broad spectrum antibiotics were discontinued. By day 4, mucositis began to improve to the point that the patient could tolerate a liquid diet. Pancytopenia persisted, however, with a platelet nadir of 49,000/uL, and leucovorin (10 mg every 6 h) was added to her regimen on day 6. By day 7, significant improvement in mucositis had occurred, pancytopenia was resolving, and she was discharged home with leucovorin, weekly B12 injections, prednisone, insulin and antihypertensives. Four days post-discharge, the WBC count was 13,900 cells/uL, the platelet count was 688, 000/uL (Table 1), and the patient had resolution of all her symptoms. Subsequently, prednisone was discontinued and etanercept was started after a negative purified protein derivative (PPD) skin test. No complications occurred during ten months of follow-up. The macrocytosis persisted with an MCV of 105, likely due to inadequate vitamin B12 replacement, as a recent vitamin B12 level was only 248 pg/ml. In follow-up, the patient reported satisfaction with her new treatment and denied adverse effects.

## Discussion and conclusions

The most common side effects of low dose MTX include fatigue, nausea, abdominal discomfort, diarrhea, stomatitis, and rash, which are usually reported in the ambulatory setting [3]. Folic or folinic acid supplementation is linked to lower rates of hepatotoxicity, gastrointestinal toxicity, and possibly to lower rates of mucosal adverse effects [1, 3, 5]. Rarely, low dose MTX may provoke severe complications such as hepatotoxicity, severe mucositis, neurotoxicity, and myelosuppression, requiring hospitalization [1, 3, 7]. Pancytopenia and oral mucositis were the most common findings in two recent studies of hospitalized patients with low dose MTX toxicity [2, 10]. MTX levels do not correlate with the clinical manifestations in low dose MTX toxicity cases [2, 10].

Reported factors increasing low dose MTX toxicity risk include acute kidney injury, drug interactions, use of other DMARDs, dosing errors, hypoalbuminemia, alcohol use, lack of folate supplementation, and obesity [1–3]. Potential contributory factors to low dose MTX toxicity identified in our patient were illiteracy, concomitant use of sulfasalazine, amoxicillin clavulanate and allopurinol, obesity, acute kidney injury, and Vitamin B12 deficiency.

Dosing errors leading to low dose MTX toxicity occurred commonly in studies of hospitalized patients [2, 10]. Physicians must strive to educate patients thoroughly regarding the *weekly* dosing of MTX and differentiate this from the *daily* folate dose [10]. Though illiterate, our patient reported the dose and interval of MTX, knew not to increase the dose in case of a flare, and used a pill box filled by a medical assistant. Hence, a dosing error appeared unlikely as a causal factor in this case.

Sorting out the contribution of comorbidities and drug interactions to low dose MTX toxicity becomes a confusing enterprise, particularly in older complex patients. According to older literature from the 1990s and early 2000s, specific comorbidities may predispose to particular types of adverse effects (reviewed by Sparks et al. 2017) [8]. Obesity, metabolic syndrome, diabetes, hypercholesterolemia, liver disease, alcohol abuse, and lack of folate supplementation have been associated with the risk of transaminitis [4, 5, 11]. The development of pancytopenia has been associated with low RBC folate, elevated mean corpuscular volume (MCV) due to folate depletion, impaired renal function, hypoalbuminemia, and infection [4, 5, 11]. Our patient presented with an MCV of 105 which we initially attributed to MTX itself, but she turned out to have another possible cause of macrocytosis-- pernicious anemia. Stopping the offending MTX likely led to the resolution of pancytopenia, as did the eventual addition of leucovorin, perhaps, but the vitamin B12 repletion may have improved matters as well. Concurrently, we had stopped SSZ and allopurinol therapy, as they also may cause cytopenias. The exact cure of pancytopenia, in this case, remains somewhat obscure.

The patient presented with acute kidney injury superimposed on chronic kidney disease. Kidney injury may have been a result of MTX toxicity, from volume depletion related to mucositis, but also may have been a cause, by raising MTX levels, as MTX is renally excreted. Allopurinol may have contributed to renal injury, thereby causing decreased clearance of MTX and increased toxicity which has been reported more frequently in the setting of high dose MTX and tumor lysis syndrome [12]. Decreased renal function has been associated with an increased risk of severe toxicity and with higher overall side effects [10, 13].

Many commonly used drugs, such as aspirin, non-steroidal anti-inflammatories (NSAIDS), proton pump inhibitors, penicillins, and trimethoprim sulfamethoxazole, interact with MTX and its levels by decreasing renal clearance [10, 14]. Our patient had recently completed a course of amoxicillin clavulanate which likely did causally contribute to the MTX toxicity.

Combination of MTX with a second DMARD may improve disease control but increase the risk of toxicity. A superior efficacy to toxicity ratio has been observed when combining MTX with sulfasalazine or hydroxychloroquine rather than azathioprine or cyclosporine [15]. SSZ was added to the patient’s regimen four months earlier. In vitro studies have demonstrated that SSZ is a non-competitive inhibitor of the reduced folate carrier which allows folate and MTX transport across the cell membrane, and therefore, hypothetically, could reduce MTX effectiveness or cause a lack of synergistic effects [16]. However, in vivo clinical trials have shown greater effectiveness of the MTX-SSZ combination than either of the drugs alone [17]. Case reports of severe pancytopenia associated with long-term SSZ use for inflammatory bowel disease noted severe folate deficiency in these patients. Our patient received a much lower dose of SSZ for a much shorter period and displayed an elevated serum folate level on admission. The high serum folate level in our patient suggests that SSZ was not the culprit of her pancytopenia [18, 19]. Red blood cell folate was not measured in our patient. However, there is no accurate evidence regarding the reliability of red blood cell folate compared to serum folate in patients on the combination of SSZ-MTX. Homocysteine, also not measured in our patient, would likely have been elevated due to concomitant vitamin B12 deficiency. Haagsma et al. randomized early RA patients to SSZ, MTX, or a SSZ-MTX combination and studied plasma homocysteine levels to measure the influence of each therapy on folate related cellular metabolism. They observed a persistent increase in homocysteine concentration in patients treated with MTX alone or the SSZ-MTX combination, in contrast to SSZ alone. They concluded that SSZ is not an efficient folate antagonist in vivo [20]. It remains possible, though less likely, that SSZ contributed to the pancytopenia in our patient via an idiosyncratic reaction.

No clear association exists between pernicious anemia and rheumatoid arthritis in the medical literature though RA has been associated with other autoimmune conditions. Anemic patients with rheumatoid arthritis most commonly exhibit anemia of chronic disease or iron deficiency anemia [21]. In a small but extensive study, vitamin B12 deficiency was discovered in 29% of RA patients but Schilling tests were normal in 80% [21]. Our patient had macrocytic indices which had developed during MTX treatment as often occurs due to impaired nucleic acid metabolism. Once vitamin B12 deficiency was confirmed in our patient, we suspected metformin as a culprit for the vitamin B12 deficiency, but ultimately the patient was found to have intrinsic factor antibodies, a specific finding of pernicious anemia [22]. Macrocytic anemia or macrocytosis without anemia should not simply be attributed to DMARDS without checking vitamin B12 and folate levels.

Vitamin B12 deficiency disrupts the folate cycle, and folate becomes trapped in an unusable form. This limits the amount of the form of folate (methylenetetrahydrofolate) necessary for the synthesis of thymidine and DNA. Tissues with rapid cell turnover, such as the hematopoietic system, are particularly affected by vitamin B12 deficiency. Erythroid precursors and granulocyte precursors malfunction, giving rise to macrocytes and hypersegmented neutrophils. Less commonly, pancytopenia may ensue. Our patient’s pancytopenia may have been accentuated by her vitamin B12 deficiency [23–25].

As noted earlier, the mechanism of action of low dose MTX remains unclear. One postulated mechanism suggests that MTX may work by interference with methyl donation [9]. MTX antagonizes dihydrofolate reductase and thereby reduces the availability of the main cellular methyl donor in humans, S-adenosylmethionine (SAM). Vitamin B12 deficiency leads to impaired methylation and results in accumulation of homocysteine and reduced synthesis of methionine and SAM [23–25]. Theoretically, the combination of vitamin B12 deficiency and MTX use could compound the antagonism of methyl donation through lower levels of SAM. We found no data on enhanced toxicity of MTX in the setting of vitamin B12 deficiency.

Our report has several limitations. We did not measure RBC folate levels which could have possibly been affected by SSZ administration. We relied, instead, on a serum folate level which was elevated and presumed the patient did not have significant folate deficiency. Nevertheless, we treated the patient with folic acid from the start and switched to folinic acid on day 6. Though we ordered a MTX level, none was performed. In studies of low dose MTX toxicity, serum MTX levels have not correlated with clinical toxicity, specifically with neither neutropenia nor thrombocytopenia [10]. We did not measure a RBC MTX polyglutamate level, which might have provided interesting information, but would not likely have changed our actions in this case.

This case illustrates the multiple factors that may potentially contribute to low dose methotrexate toxicity by increasing serum MTX levels or interfering in its metabolic pathway. Our analysis sheds light on the difficulty of assigning causality to any one factor. It highlights the importance of testing for B12 deficiency in RA patients with macrocytic anemia as knowledge of vitamin B12 deficiency may play a role preventing some complications associated with folate antimetabolite medications. Evaluation of all the potential contributors to low dose methotrexate toxicity in the outpatient setting may help avoid hospitalization and improve patient outcomes.

### Patient perspective

I remember I went to the hospital because the pain in my mouth was awfully bad and I could not take even a sip of water. I did not look for medical attention very soon cause at the beginning I thought it was a viral infection or so. I am glad about the results by the time I left the hospital, the treatment worked very well. I think the methotrexate was a good medication. I knew from my doctor that side effects are common and that when they occur, it is better to stop the medication and to find medical attention, but I just did not think it could be that serious. Now, my rheumatoid arthritis is well controlled on Enbrel and so far, I have not had any issues. Thank you for taking care of me and I really hope the paper helps other people.

## Data Availability

N/A

## Abbreviations

DMARDS: Disease modifying antirheumatic drugs
MCV: Mean corpuscular volume
MTX: Methotrexate
RA: Rheumatoid arthritis
